# Functional Reversal as a Feasible Option for Malnutrition in Patients With Hyper-Response Following Roux-en-Y Gastric Bypass: A Case Report

**DOI:** 10.7759/cureus.99614

**Published:** 2025-12-19

**Authors:** Martha P Sánchez Muñoz, César A Ortiz Orozco, José D Reyes Blandón, Carlos M Moreno Mendoza, Soledad Aldana Aguiñaga

**Affiliations:** 1 Bariatric and Metabolic Surgery, Hospital Civil de Guadalajara Dr Juan I Menchaca, Guadalajara, MEX

**Keywords:** bariatric & metabolic surgery, bariatric surgery, gastric bypass reversal, malnutrition, obesity

## Abstract

Obesity is recognized as a global epidemic, and bariatric surgery is the most effective treatment for obesity and its associated comorbidities, providing durable long-term outcomes. However, a subset of patients develops malnutrition as a complication despite adequate nutritional supplementation, for whom revisional surgery may be indicated, although such procedures can be technically and clinically challenging.

We discuss the case of a 55-year-old female with a significant surgical history, including three cesarean sections and a fundoplication performed 17 years earlier, who underwent Roux-en-Y gastric bypass (RYGB) at a private hospital. At the time of bariatric surgery, her weight was 107 kg, with a BMI of 41.7 kg/m² and a height of 1.60 m. One year and nine months postoperatively, she presented to our center for the first time, reporting generalized weakness, asthenia, and reduced physical activity, despite adherence to prescribed nutritional supplementation and dietary recommendations. After a complete assessment and laboratory tests identifying normocytic normochromic anemia and severe malnutrition (albumin 2.5) and one month of mixed nutrition, we performed revisional surgery with functional reversal of RYGB with later resolution of her nutritional deficiencies.

The RYGB reversal using Gastro-gastric anastomosis (functional reversal) appears to be a feasible, less complex option with a potentially lower complication risk than full anatomical reversal in malnourished RYGB patients.

## Introduction

Bariatric and metabolic surgery is currently regarded as the primary treatment for obesity and its associated metabolic comorbidities, offering durable long-term outcomes. The most commonly performed bariatric procedures worldwide are Roux-en-Y gastric bypass and laparoscopic sleeve gastrectomy [1]. The anatomical alterations resulting from RYGB lead to reduced nutrient absorption, which is generally managed through appropriate supplementation and regular nutritional follow-up. However, a small proportion of patients develop nutritional complications despite adequate nutritional follow-up. As the prevalence of bariatric surgery increases, nutritional follow-up and patient adherence to nutritional supplementation guidelines will be increasingly critical to ensuring long-term surgical success [1].

Nutritional complications after bariatric surgery that may necessitate revisional surgery include severe malnutrition, refractory dumping syndrome, persistent hyperinsulinemic hypoglycemia, anemia, and inadequate weight loss, among others [2]. Management of these complications can be challenging, and in some cases, reversal to normal anatomy with the restoration of pyloric and duodenal transit represents a definitive therapeutic option [3].

## Case presentation

A 55-year-old businesswoman, with a medical history of obesity, arterial hypertension, and insulin resistance, and a surgical history of three cesarean sections and a fundoplication performed 17 years ago, underwent RYGB at a private hospital. At the time of bariatric surgery, her weight was 107 kg, height 1.60 m, and BMI 41.7 kg/m². One year and nine months after surgery, the patient consulted our center for the first time, reporting generalized weakness, asthenia, and adynamia, despite adherence to supplementation and nutritional recommendations, as she described. A complete assessment and laboratory workup revealed normocytic normochromic anemia and severe malnutrition (albumin: 2.5 g/dL).

The patient was evaluated by a transdisciplinary team, ruling out psychiatric or psychological disorders. From a surgical perspective, an upper gastrointestinal endoscopy was performed to rule out structural abnormalities. The examination revealed a normal esophagus, a 5-cm gastric pouch, a 2-cm anastomosis without pathological findings, and a candy cane limb with a normal appearance. Initially, conservative management was chosen with appropriate nutrition and supplementation to improve her nutritional status.

However, her symptoms progressively worsened. At that time, her weight had decreased to 66.9 kg, with a BMI of 26 kg/m², and laboratory studies demonstrated a hemoglobin level of 8.59 g/dL, hematocrit of 27.7%, albumin of 1.4 g/dL, and total protein of 3.6 g/dL. She was hospitalized for mixed (enteral/parenteral) nutrition and evaluation for revisional surgery. After one month of hospitalization, an improved nutritional status was achieved. Therefore, a diagnostic laparoscopy with revision of the gastric bypass was performed.

Surgical technique

Under general anesthesia, with the patient positioned in the American position, the abdomen was prepared and draped in a sterile fashion. A 12-mm optical trocar was placed in the left upper quadrant using the Hasson technique, followed by placement of 12 mm, 11 mm, and 5 mm trocars in the right and left upper quadrants. Diagnostic laparoscopy revealed anatomical findings consistent with RYGB, along with firm adhesions between intestinal loops and the abdominal wall, which were released using the LigaSure™ Maryland energy device until the gastrojejunal anastomosis was clearly identified.

The small intestine was completely measured, yielding the following lengths: an alimentary limb of 200 cm, a biliopancreatic limb of 260 cm, and a common limb of 830 cm. These findings corresponded to a total alimentary limb length (TALL) > 400 cm, suggesting that the malnutrition was not due to anatomical causes but likely a hyper-response to the RYGB. Therefore, a functional reversal of the bypass was performed via a gastro-gastric anastomosis.

**Figure 1:**
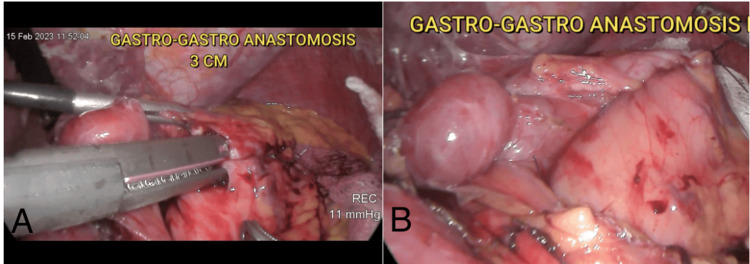
Intraoperative images A) 3-cm gastro-gastric anastomosis with Tri-Staple linear stapler. B) Completed anastomosis closed in two layers with Monocryl 2-0

Adhesiolysis was performed at the pouch and the most proximal portion of the native stomach. Reference sutures were placed with Monocryl 2-0, followed by gastrotomies on the posterior aspect of the pouch and the anterior aspect of the native stomach. A 3-cm gastro-gastric anastomosis was created using a Tri-Staple™ 3.5 x 60 mm purple cartridge (Medtronic, Minneapolis, MN). The anastomosis was closed in two layers using Monocryl 2-0 (Figures 1A, 1B).

A methylene blue test showed no evidence of leakage. The procedure concluded with placement of a drainage tube, removal of surgical materials, and hemostasis. The total surgical time was 150 minutes, with an estimated blood loss of 50 mL.

**Figure 2:**
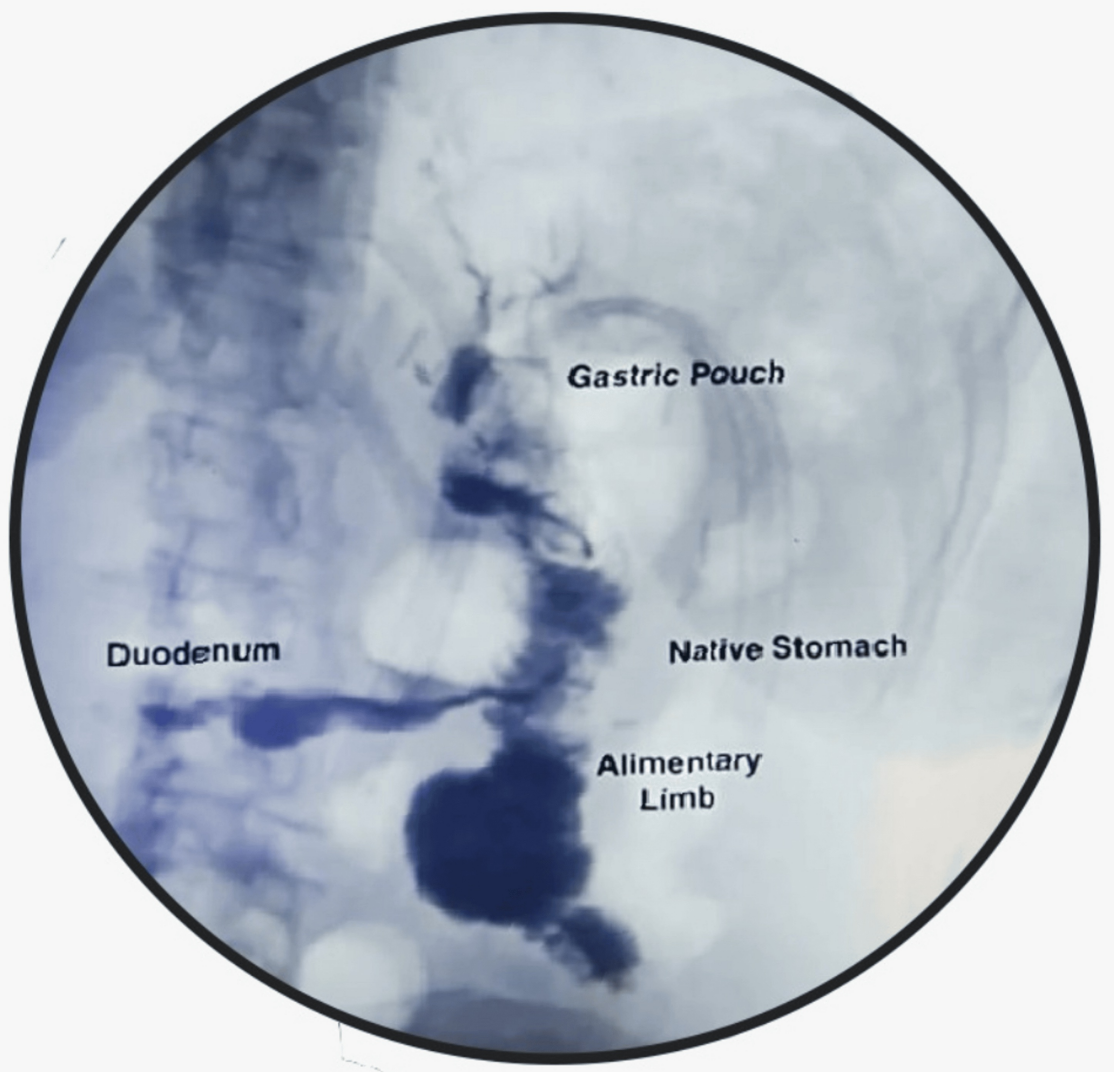
Postoperative image - esophagogram on postoperative day one The image demonstrates appropriate transit of contrast medium through the jejunum, native stomach, and duodenum, without any evidence of anastomotic leak

The patient had an uneventful recovery. A water-soluble contrast study performed the next day showed adequate passage of contrast into the alimentary limb, native stomach, and duodenum without any leaks (Figure 2). The patient was discharged the following day with nutritional recommendations.

Three months following the revisional surgery, the patient demonstrated complete resolution of symptoms, with a body weight of 72 kg, a BMI of 28 kg/m², and normalization of laboratory values (Table 1).

**Table 1:** Evolution of laboratory results The values in parentheses represent the reference ranges

	Hemoglobin (12-16 g/dl)	Hematocrit (38-47%)	Platelets (150-400 10^3^/ µL)	Albumin (3.5-5.4 g/dl)	Total proteins (6.4-8.3 g/d)	Iron (35-145 mcg/dl)	Ferritin (13-232 ng/ml)	Glucose (70-100 mg/dl)
Initial consultation	10.6	32	303	2.8	5.5	60	154	50
Pre-hospitalization	8.59	27.7	737	1.4	3.6	105	390	56
Pre-surgical	9	29.02	479	3.3	5.9	100	279	78
Post-surgical	12.38	38.49	311	4.40	6.7	76	137	81

## Discussion

The number of bariatric surgeries performed worldwide continues to grow, and consequently, the number of patients requiring revisional procedures is also anticipated to rise. These revisional surgeries are often technically demanding and require individualized assessment and a high level of surgical expertise to achieve favorable outcomes with minimal morbidity and risk to the patient [4]. In 2006, Himpens et al. published the first laparoscopic reversal of RYGB to normal anatomy in a 46-year-old female patient with refractory dumping syndrome [5]. Since then, various surgical techniques have been described to restore normal anatomy after bariatric procedures. However, there is no established consensus on the optimal technique, and the decision must be individualized based on patient characteristics and surgical findings [3,6].

The primary indications for gastric bypass reversal include severe dumping syndrome, hyperinsulinemic hypoglycemia, refractory marginal ulcers, and severe malnutrition [2]. It is well established that one of the mechanisms by which bariatric surgery promotes sustainable weight loss is through reduced nutrient absorption. Given that RYGB is the most widely used malabsorptive procedure, a certain proportion of patients are expected to develop nutritional deficiencies, an outcome that has been widely reported and documented in the literature [7]. To prevent such complications, adequate micronutrient and macronutrient supplementation is essential in the long term [8].

Among nutritional deficiencies, protein deficiency is most commonly associated with postoperative malnutrition. Current recommendations suggest a daily protein intake of 60-120 grams for bariatric patients, particularly those who have undergone malabsorptive procedures [9]. Therefore, poor adherence to dietary and supplementation guidelines can easily lead to malnutrition. In our patient’s case, however, malnutrition occurred despite adequate adherence to nutritional protocols. Mixed enteral and parenteral supplementation was required to improve her nutritional status. Diagnostic laparoscopy revealed relatively normal post-RYGB anatomy, supporting the decision to proceed with gastric bypass reversal.

Existing literature on gastric bypass reversal is limited, primarily comprising case reports that describe a range of surgical techniques. In 2018, López et al. reported a case of RYGB reversal in a 51-year-old malnourished patient using the Branco-Zorrón technique, which involves transecting the alimentary limb at the jejunojejunostomy and anastomosing it to the native stomach, an approach noted for its low morbidity and favorable outcomes [10]. In one of the largest published series, Ma et al. (2019) reported their experience with gastric bypass reversal in 46 patients treated between 2012 and 2016 at a single institution. Of these, 48% underwent reversal due to malnutrition, although specific techniques used were not described. The overall 30-day complication rate was 29%, including anastomotic leaks, bleeding, and readmissions, with a 30-day mortality rate of 2% [11]. In 2020, Shah and Gislason introduced a new approach called "functional reversal", which involves the creation of a gastro-gastric anastomosis while preserving the RYGB anatomy. This technique was proposed to be technically less complex, associated with less weight regain, and equally effective in symptom resolution compared to full reversal to normal anatomy [12].

In our case, we chose to perform a functional reversal via gastro-gastric anastomosis, given the patient’s advanced age, surgical risk, nutritional condition, and desire to avoid weight regain. This technique allows part of the food bolus to resume its physiological passage through the duodenum and jejunum, thereby enhancing nutrient absorption. It emulates the principle of intestinal bipartition described by Santoro et al. [13]. At five months postoperatively, the patient remains symptom-free, with optimal nutritional status and normal laboratory values, supporting the conclusion that gastro-gastric anastomosis is a safe and effective option for the treatment of severe malnutrition following gastric bypass.

## Conclusions

RYGB reversal using gastro-gastric anastomosis (functional reversal) is a feasible and less complex option and is apparently associated with a lower risk of complications compared to full reversal to normal anatomy in patients with malnutrition after RYGB. However, further studies involving longer follow-up and larger sample sizes are needed to confirm these findings and provide stronger evidence. It is important to note that patients who undergo reversal of any bariatric or metabolic procedure are at risk of weight regain. Therefore, close monitoring is essential, including regular follow-up with the bariatric surgeon every 6-12 months, as well as ongoing nutritional and psychological counseling. This multidisciplinary approach helps to detect and prevent weight regain while reinforcing adherence to lifestyle and dietary interventions as needed.
